# “Contraceptives are like committing a crime”: a qualitative study on contraceptive access and utilization in Libya

**DOI:** 10.3389/fgwh.2026.1776991

**Published:** 2026-06-24

**Authors:** Mariam Omar Elsaltani, Angel M. Foster

**Affiliations:** 1 Faculty of Health Sciences, University of Ottawa, Ottawa, ON, Canada; 2 Faculty of Public Health, University of Benghazi, Benghazi, Libya; 3 Collaborative for Interdisciplinary Global Abortion Research, Ottawa, ON, Canada

**Keywords:** contraceptive access, family planning, healthcare providers, Libya, reproductive health, social norms, structural barriers, women's health services

## Abstract

**Introduction:**

Contraceptive access is a critical component of sexual and reproductive health and rights, as well as an indicator of health system performance and broader commitments to gender equity. While global frameworks like Family Planning 2030 emphasize the need for equitable and rights-based access to contraception, Libya's centralized yet fragmented health system, compounded by political instability and weak national monitoring, has contributed to gaps in contraceptive service delivery and limited understanding of how women navigate access in practice. This study examines women's contraceptive decision-making in Libya, and the social, political, and institutional factors that shape women's access to and use of contraceptive methods.

**Methods:**

We conducted a multi-method qualitative study in 2023–2024 in three major cities in Libya: Tripoli, Benghazi, and Sabha. We conducted 17 in-depth interviews with women of reproductive age, 9 focus group discussions with 60 women, and 11 key informant interviews with healthcare providers and program managers. We analyzed these data sources for content and themes using an iterative approach.

**Results:**

Three intersecting themes emerged: 1) Intense societal and familial pressure to bear children, especially sons, exists; 2) The politicization and moral scrutiny of contraception in Libya leads to conditional and uneven access; and 3) Women covertly navigate contraceptive use as a result of risk and stigma. Contraception was more socially tolerated when framed through marriage, spacing, or religiously acceptable purposes, while other forms of use were more heavily scrutinized. Despite persistent barriers, some women described making pragmatic reproductive decisions in response to economic hardship and ongoing instability.

**Discussion:**

Efforts to improve contraceptive access in Libya must move beyond availability toward inclusive, system-strengthening, and rights-based approaches that acknowledge normative and structural barriers. Our findings show that in Libya, contraceptive access is shaped not only by health system gaps, but also by the conditional social and political acceptability of contraception.

## 1 Introduction

Contraceptive access is recognized globally as a cornerstone of sexual and reproductive health and rights and critical to advancing gender equity, improving maternal health outcomes, and supporting broader public health goals (1). Access to and use of contraception are recognized as indicators of a country's health system performance and its broader commitments to gender equity and human rights (2). Global commitments such as the International Conference on Population and Development (ICPD) and Family Planning 2030 (FP2030) highlight the importance of equitable and reliable access to a range of contraceptive methods as part of universal health coverage as well as sustainable development (3, 4).

Libya, classified as an upper-middle-income country, has long offered free healthcare and education to its citizens (5–7). However, years of protracted conflict, political fragmentation, and underinvestment have severely undermined the health system's ability to meet population needs (8). Although 85% of Libya's population lives in urban areas, the country remains one of the most sparsely populated in the world, complicating health service delivery (9). The public health system is highly centralized under the Ministry of Health but operates across more than 100 municipalities, each tasked with health planning and implementation (8). This overlapping governance structure has resulted in inconsistent service quality and delivery (8). While Libya has a relatively high density of health workers nationally (7.6 per 1,000), services are heavily physician-centric, with limited integration of mid-level providers and weak regulatory oversight of the expanding private sector (10). Human resources are unevenly distributed, with some regions understaffed and others overstaffed. Health infrastructure remains uneven, especially in the south, where physical access, provider presence, and availability of supplies are limited (8, 10).

Sexual and reproductive health (SRH) services, particularly contraceptive provision, are among the most affected by systemic constraints (11). Reliable national data on contraceptive availability and uptake in Libya remain limited. Much of what is known stems from sporadic international assessments or localized programmatic reports. According to World Bank estimates, Libya's total fertility rate currently is 2.4 children per woman, lower than the North African regional average (approximately 3.0) and roughly in line with the current global average (about 2.3–2.4) (12). Despite this moderate fertility level, the contraceptive prevalence rate remains low at 27.7%, with an estimated unmet need of at least 40%, among the highest in the region (10). Available evidence suggests that contraceptive services in public health facilities are limited, with SRH care highly medicalized and focused primarily on consultations. Nurses and midwives play a minimal role in family planning provision, and adolescent- and youth-friendly services are largely absent (13). Although Libya's 2019 Reproductive, Maternal, Newborn, Child, and Adolescent Health (RMNCAH) strategy aimed to strengthen SRH integration, implementation has been minimal (10). Frequent stockouts, insufficient provider training, and lack of integration of SRH into primary care reflect a broader pattern of system deficiencies. Humanitarian organizations strive to fill essential gaps, particularly for displaced populations, but their reach is limited and long-term sustainability is uncertain (13).

Against this backdrop of weak service provision and limited national monitoring of national and international commitments, there remains a critical evidence gap around women's lived experiences with contraception in Libya. Quantitative indicators offer only a partial view; they fail to capture how women make decisions, what barriers they face, and how they navigate contraceptive use in practice. While global frameworks often define contraceptive access through metrics of availability and unmet need, such measures risk oversimplifying the structural and normative frameworks that govern reproductive life in Libya. This gap is especially important in Libya, where contraceptive access must be understood within a health system shaped by political fragmentation, inconsistent service delivery, and broader social expectations surrounding women's reproductive roles. We aimed to explore this gap through a multi-method qualitative approach, capturing the perspectives of women and key informants involved in the delivery, coordination, or oversight of reproductive health services across urban Libya. We sought to examine how contraceptive decision-making and access are shaped not only by service availability, but also by broader socio-political norms and institutional dynamics**.** This study contributes to global understanding of contraceptive access by examining how access is shaped not only by service availability, but also by broader institutional, social, and political conditions.

## 2 Methods

Between September 2023 and late 2024, we conducted a multi-method qualitative study as part of a larger research project examining women's experiences with SRH and gender-based violence services in Libya. For this study, we specifically explored how contraceptive decision-making and use are experienced, navigated, and shaped in the Libyan context. We grounded our approach in the framework of population health, which emphasizes the structural and systemic determinants of health outcomes (14, 15) and in reproductive justice principles, which center the right to bodily autonomy within intersecting systems of oppression and privilege (16).

Drawing on the Minimum Initial Service Package for SRH in Crisis Situations (17) and the Inter-Agency Field Manual on Reproductive Health in Humanitarian Settings (17), and building on previous needs assessments our team conducted in similar fragile contexts, including Albania, Jordan, Myanmar, Somalia, and Uganda (18–22), we sought to answer several interrelated questions: How do women navigate contraceptive decision-making and utilization? What social, political, and institutional factors shape women's access to and use of contraceptive methods? And, how providers and key informants perceive the challenges influencing contraceptive service delivery and uptake?

### 2.1 Study population and setting

**Figure 1:**
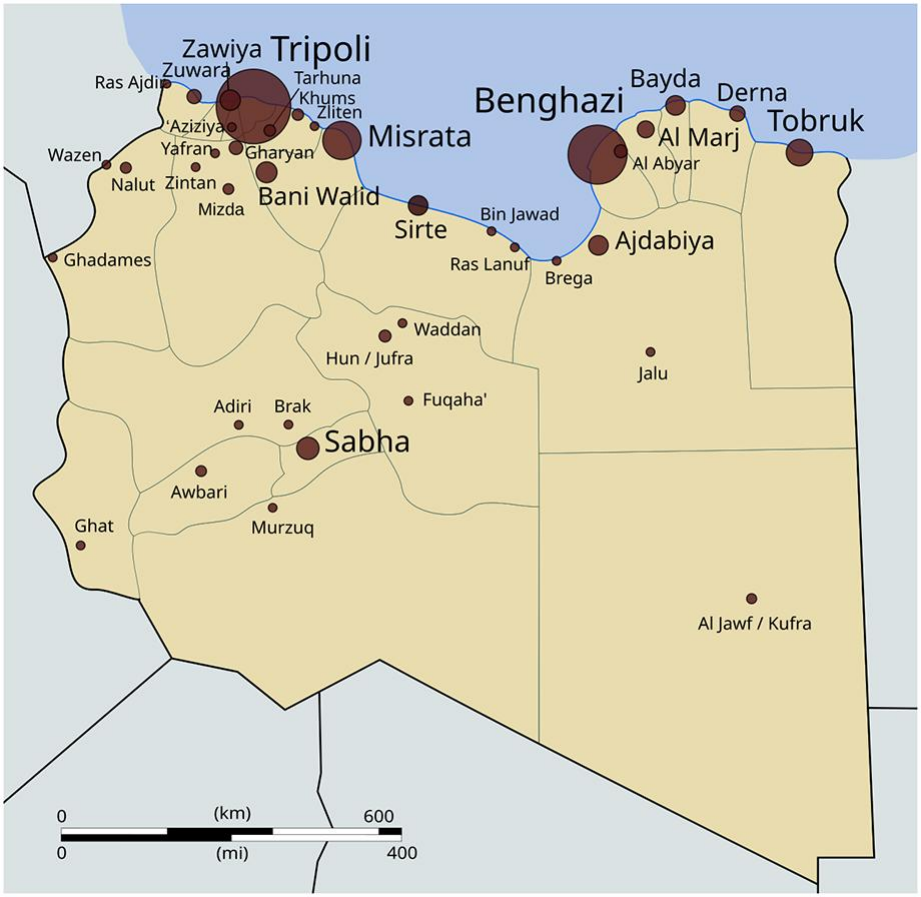
Map of Libya showing the three study cities: Tripoli (west), Benghazi (east), and Sabha (south). Source: Wikimedia Commons, File: Libyan_Uprising.svg License: Creative Commons Attribution-Share Alike 4.0 International (CC BY-SA 4.0) We have reproduced this map without adaptation. Attribution: Wikimedia Commons contributors, CC BY-SA 4.0, via Wikimedia Commons.

We recruited women and key informants from three urban centers: Tripoli, Benghazi, and Sabha, representing Libya's Western, Eastern, and Southern regions (see Figure 1). Although these are not official administrative divisions, they align with Libya's historic provinces of Tripolitania, Cyrenaica, and Fezzan, which originated during the Ottoman era (23) and continue to shape political and humanitarian programming today. This approach allowed us to capture regional diversity in women's experiences.

MOE, a Libyan PhD candidate in Population Health at the University of Ottawa at the time of the study, led the data collection after receiving training and supervision from AMF, a medical anthropologist and medical doctor with extensive experience in SRH research in fragile and humanitarian contexts.

### 2.2 Data collection

#### 2.2.1 In-depth interviews

We conducted 17 semi-structured interviews with women of reproductive age (15–49 years) recruited through local non-governmental organisations (NGOs), community organizations, and early participant referrals. We held interviews, which lasted between 60 and 90 min and were audio-recorded with consent, in Arabic. MOE took detailed field notes and memos immediately following each session. Interviews explored women's contraceptive decision-making as well as their experiences seeking and using contraceptive services and perceived barriers to accessible care.

#### 2.2.2 Focus group discussions

MOE facilitated 9 focus group discussions (FGDs) with women across the three cities (60 women in total). To maintain a degree of social homogeneity and reduce power differentials, we stratified FGDs by marital status (married vs. unmarried women). Discussions, conducted in Arabic and moderated by MOE with assistance from trained local research assistants, centered on women's perceptions of service availability, societal norms, and barriers to contraceptive access. Recruitment occurred through women's organizations, networks, and word-of-mouth.

#### 2.2.3 Key informant interviews

MOE conducted 11 interviews with key informants, including healthcare providers, NGO workers, humanitarian program managers, and government-affiliated staff. We identified key informants via public records, project contacts, and early participant referral. Interviews explored informants' professional experiences with delivering or coordinating SRH services, perceived barriers to contraception provision, and observed changes in community attitudes toward family planning/contraception.

### 2.3 Data management and analysis

We audio-recorded and transcribed all interviews and FGDs verbatim. MOE translated all transcripts from Arabic into English for analysis. We employed an iterative analytic approach, using continuous comparison and memoing throughout the study period. Coding combined deductive strategies based on study aims and inductive techniques to capture emergent themes. We managed our data using ATLAS.ti. MOE conducted initial coding and AMF contributed to theme refinement and interpretation. We first analyzed data separately by component (in-depth interviews, FGDs, key informants) and then triangulated across sources to identify convergent and divergent findings.

### 2.4 Ethics

The study received ethics approval from the University of Ottawa's Social Sciences and Humanities Research Ethics Board (File #: S-08-18-1029) and the University of Benghazi's Research Ethics Committee. All participants provided informed consent. We have used pseudonyms and removed identifiable information to protect participant confidentiality.

## 3 Results

### 3.1 Participant characteristics

Our sample of 17 in-depth interview participants included women spanning the full reproductive age range and reflected diversity in educational attainment and marital status. As shown in Table 1, over half of the participants were under 30 years old (*n* = 9, 53%), with the largest subgroup aged 18–24 (*n* = 5, 29%). The geographic distribution included Benghazi (*n* = 7, 41%), Tripoli (*n* = 6, 35%), and Sabha (*n* = 4, 24%). Notably, two participants based in Benghazi had previously accessed services in Tripoli, allowing them to comment on regional differences in service availability.

**Table 1:** Demographic characteristics of in-depth interview participants from three Libyan cities (*N* = 17).

Characteristics	*n*	%
Age (years)		
18–24	5	29%
25–29	4	24%
30–40	3	18%
41–45	2	12%
46+	3	18%
Level of education		
Middle School Certificate	1	6%
High School Diploma	2	12%
Bachelor's Degree	10	59%
Graduate Degree	4	24%
Employment		
Employed	10	59%
Non-employed	7	41%
Relationship Status		
Single	5	29%
Married	9	53%
Separated/Divorced/Widowed	3	18%
Location		
Benghazi	7	41%
Tripoli	6	35%
Sabha	4	24%

With respect to education, most participants held at least a bachelor's degree (*n* = 10, 59%), and four reported having completed graduate studies (24%). A majority were employed at the time of the interview (*n* = 10, 59%); the remainder were not employed (*n* = 7, 41%). In terms of relationship status, just over half were married (*n* = 9, 53%), five were single (29%), and three were separated, divorced, or widowed (18%). Our FGDs engaged 60 women aged 15–49 across marital statuses. Table 2 outlines the demographic composition of the FGD participants.

**Table 2:** Demographics of focus group discussion participants by age, marital status, and geographic location.

Group	Number of participants	Average age	Marital status	Geographic location
1	8	20	Unmarried	Benghazi
2	6	20	Unmarried	Benghazi
3	8	41	Married	Benghazi
4	6	44	Married	Tripoli
5	6	34	Unmarried	Tripoli
6	6	44	Married	Tripoli
7	5	35	Married	Sabha
8	5	42	Married	Sabha
9	10	26	Unmarried	Sabha

We also conducted interviews with 11 key informants based in Tripoli (*n* = 3), Benghazi (*n* = 5), and Sabha (*n* = 3). Many informants had cross-institutional experience with organizations involved in delivering or coordinating SRH services. Five informants were affiliated with public sector institutions, four were employed in the private sector or by NGOs. The group included four physicians or direct service providers, two coordinators from the Institute of Public Health (including one overseeing pharmaceutical logistics), two NGO representatives involved in SRH programming, one pharmacist and two KIs whose roles spanned more than one institutional or service category. This institutional diversity offered a broad view of the SRH service landscape across the three cities.

Drawing on triangulation across in-depth interviews, FGDs, and KIIs, we present below three interrelated themes. While the themes were broadly consistent across all three components, each data source contributed a distinct perspective. In depth interviews provided more detailed accounts of women's lived experiences and decision making, FGDs highlighted shared norms and collective expectations, and KIIs offered institutional and service delivery perspectives.

### 3.2 Societal pressure to become mothers is intense

Participants in interviews, FGDs, and key informant discussions across all regions described intense societal and familial pressure on women to become mothers early, repeatedly, and often in pursuit of sons. This pressure was not only emotional but deeply structural, tied to marriage, womanhood, and social legitimacy. In this context, contraceptive use was often seen as a deviation from one's expected reproductive role.

Fatma, 27, from Benghazi, explained that the pressure to become pregnant begins almost immediately:

The society, they ask you, did you get pregnant? When will you have a child? A boy? A girl? The social pressure is hard on the women…I was forced to have the second child so I can belong and continue. I feel I wasn't in my whole mind-set…and I realize now that I shouldn't have done that.

This sentiment was echoed by many participants, several of whom described becoming pregnant not out of desire, but obligation. Providers confirmed these dynamics. One key informant noted, “You'll see a woman with a 4-month-old baby and she's already trying to get pregnant again…her husband told her to keep going, have two kids back-to-back, then take a break.”

Across interviews and FGDs, having three or more children was described as the baseline social expectation. “A woman must have at least three kids. This is what society tells her,” said Rania, 45, from Sabha. “Even four children are not enough,” added Abeer, 29, also from Sabha. Participants also shared experiences of public humiliation when those expectations weren't met: “Only three? That's not much…That woman, you always see her either pregnant or having just given birth.”

Family pressure, particularly from in-laws, was another common theme. Majdoleen, 26, from Benghazi, recalled: “My mother-in-law placed children in my lap and said, ‘Maybe this will get you pregnant.’” Women who had not given birth to sons faced especially acute stigma. One participant noted, “Whenever a woman gives birth to a girl she goes through a whole lot of abuse and bullying.” Fathia, 40, from Benghazi, shared that her mother-in-law once warned: “If you are pregnant with a girl, pack your bag.” A provider elaborated: “Culture-wise, the male carries the family name, so it's crucial to have a large number of children. A woman is pressured to have many children even if her health doesn't allow it.” Fathia, 40, from Benghazi added: “A woman is pressured… and she is threatened with the possibility of her husband marrying someone else.”

The societal framing of womanhood as synonymous with motherhood often sidelined women's physical and mental health, with little regard for their well-being or autonomy. Aya, 27, from Tripoli, shared: “My husband keeps asking me to have another child…I'm so tired physically and mentally…I'm taking antidepressants…I can't get pregnant, but he is not understanding this.” Even postpartum, expectations continued. As another participant recalled, “My father-in-law came after a week and told me I rested for too long and asked me to do my house chores.”

Importantly, the societal framing of reproduction also shaped men's decisions. Even when a couple agreed not to have children, external judgment could override their private decisions. “Even if her husband is convinced and doesn't want children, they will fear society,” explained Fatma, 27, from Benghazi:

What will the community say about us? What will my mother say about us? My friends, my relatives?… They will say I am not man enough to have children, or that he is staying with his wife despite her not being able to have children. She is faulty. So they have to prove otherwise and have children. Even those who are educated… they are forced.

The cost of this coercion extended beyond emotional tolls. Maram, 39, from Benghazi, described how an embryo implantation, undertaken under family pressure, led to a psychological crisis: “This experience caused me many psychological problems. I didn't find support, and the hormones also started my psychological crisis.” Fatma, 27, added, “It delayed my educational progress and my career and my health. I am 27 and my health is not the health of a 27-year-old girl. I feel I am battling life like I'm in my forties.”

### 3.3 Contraceptives are both widely condemned and conditionally available

Participants across all study components explained that while some contraceptive methods are formally available in Libya through pharmacies and private clinics, access remains deeply constrained by normative expectations toward motherhood. The societal norms that demand women bear children not only discourage contraceptive use but also shape how institutions, providers, and decision-makers respond to family planning. Within this context, contraception is not only discouraged socially, but also politicized, morally scrutinized, and functionally restricted. One FGD participant captured this pervasive hostility, stating, “Contraceptives are like committing a crime.”

Fears about promoting extramarital sex, undermining morality, and weakening national identity were frequently cited. One key informant, working on a reproductive health initiative, explained how even engaging in contraception-related work could draw suspicion from government officials. In one encounter, he was asked with disbelief and discomfort, “What do you mean you provide contraception?” He elaborated that decision-makers perceived family planning as a foreign agenda designed to weaken Libyan society: “Family planning is often accused of wanting to reduce the Libyan population and replace Libyans with foreigners.” Participants explained that in government circles, contraceptives were portrayed as a morally corrupting influence, particularly for women, because they were believed to facilitate extramarital sex, which is criminalized in Libya.

This framing created a politically charged atmosphere in which both the services themselves and the language used to describe them were challenged. To prevent backlash or allegations of impropriety, key informants reported using specific terminology when delivering services or communicating with government entities. Participants described that the Arabic term for contraception, māniʿ al-ḥaml (
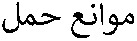
, “pregnancy preventers”), was considered inflammatory and inappropriate in many circles, as it was perceived to imply an intent to block reproduction altogether rather than to space or plan births. As one informant explained, “If we write māniʿ al-ḥamel, people think we're trying to prevent Libyan women from conceiving…but if we write ‘family planning’, it means giving families the option to space pregnancies.”

For women, this environment of suspicion translated into direct barriers to access. Providers routinely denied contraceptive services based on personal beliefs or required proof of marriage. One key informant explained, “They sell them in some pharmacies… but the doctors have to make sure the woman is actually married.” Salma, 20, from Benghazi recounted that her relative was denied services altogether: “My relative was denied contraceptives by a doctor who doesn't support family planning.” These dynamics sometimes led to women not seeking services at all, rather than risk humiliation, rejection, or suspicion.

On the other hand, contraceptives were more acceptable when tied to religious or aesthetic purposes rather than fertility management. “Contraceptives are used for fasting and Umrah [pilgrimage]…unmarried women can obtain them for reasons like fasting and travel” said Maram, 39 from Benghazi. An FGD participant explained, “Girls use it for parties or Ramadan”, and others described brides timing their periods to avoid menstruation during wedding celebrations.

Structural and economic barriers further compounded the effects of social rejection. Contraceptive access was often limited to private clinics, with public health facilities lacking supply. One provider explained, “We no longer procure supplies..they are not part of the national procurement plan.” Even humanitarian organizations faced restrictions. “The Ministry of Health has a very restricted list of supplies.”

Affordability remained a significant constraint, particularly for certain methods such as the intrauterine device (IUD). Although Hana, 40, from Benghazi, noted that oral contraceptives were “actually not expensive… because there is no demand,” access to longer-acting methods was more limited. A participant in the Sabha-based FGD reported that an IUD is “often only inserted in private clinics, and costs around 500 Libyan Dinars”, a prohibitive sum for many women. For others, financial limitations shaped their choices more directly. “I used to get the good ones [like Yasmin, a combined oral contraceptive pill widely viewed as a trusted brand]…but the price went up, and I couldn't afford them. So I started using the regular pills[cheaper, locally available generics],” explained Nesma, 43, from Tripoli. These financial barriers, compounded by moral scrutiny and uneven service provision, significantly limited women's ability to access contraception.

### 3.4 Navigating contraceptive use in secrecy

In this restrictive environment, many women turned to discreet and informal strategies to access contraceptives, often supported quietly by trusted providers. Many women in interviews or FGDs described using contraceptives “in secret” as a strategy of resistance, risk avoidance, and survival. Providers, too, recounted the need to navigate moral and political sensitivities when offering contraceptive/family planning services.

Women frequently accessed contraception without their husband's knowledge or consent. “She told me, ‘Doctor, please be careful, if my husband finds out, he'll cause problems.’” One FGD participant noted “[If] She wanted family planning without her husband's knowledge she talks to a doctor she trusts”. Another woman in the Sabha-based FGD confirmed, “I met some women in Tripoli who told me they use contraception in secret…Yes, even here, we have that too”.

This secrecy, however, carried significant personal risk. One Tripoli-based FGD participant recounted a friend who “had side effects and was taken to hospital…when he [her husband] realized it was because she used an IUD, he divorced her.” Another from Sabha shared, “A woman, caught using [oral contraceptive pills], was publicly shamed by her husband.” In response, providers developed strategies to protect their patients and themselves, including keeping women's medications in the clinic. “One patient brought me six packs of [oral contraceptive] pills… I had to pretend to do lab tests just to cover for her.”

Women covertly navigating contraceptive use were often managing abuse, control, and economic dependence. A provider shared:

She told me “I live with a man who drinks and beats me and the kids. I don't want to get pregnant from him, but I have no support. Where would I go? My brothers treat their wives the same. At least here, I'm surviving. Divorce to go where?”

These testimonies illustrate how reproductive decisions are deeply entangled with women's safety, mobility, and survival within a broader context of limited autonomy and constrained choices. Several participants described exercising agency by making firm reproductive decisions, despite the limitations imposed by their environment. “I decided early, right after the four kids, to not have more children,” shared Rania, 45, from Sabha.

Some participants noted what they perceived as a generational shift toward smaller families and reflected on how broader social and economic changes in Libya were influencing reproductive decision-making. “These years, you feel the circumstances have become difficult…there's an increase in prices for everything…you start thinking about the cost,” shared one Benghazi-based FGD participant. Another added, “After the revolution…people stopped at three kids because they say, ‘I'm displaced, I live in a rented apartment…the children have a lot of needs.’” For some, contraception became a pragmatic response to the instability of daily life. A key informant noted, “A lot of men who opposed family planning now say we need it…the economic status and health status of women convinces him of the need for family planning.” As Fatma, 27, from Benghazi, concluded, “I hope people can understand that a woman has more time to have children, and that she can still be able to get pregnant at 45.”

## 4 Discussion

In this study, we explored how women in Libya make decisions about and use contraception while navigating realities shaped by policy gaps, limited institutional support, and instability. Our findings illustrate that even where contraceptive methods are technically available, women's ability to access them is shaped by far more than supply alone. We show that contraceptive access in fragile and politically restrictive settings cannot be understood through service availability alone, but must be situated within the wider social, moral, political, and institutional conditions that shape access in practice.

Our findings suggest that in Libya contraceptive access exists at the intersection of moral policing, religious interpretations, politicized narratives, and social conformity. Decisions around contraception are not merely personal or clinical, they are shaped by existing social hierarchies and everyday power relations that reveal deeper tensions over gender and political authority. At the heart of these dynamics lies the societal expectation that women must bear children early, often, and preferably male. Reproductive capacity in Libya appears to be deeply linked to womanhood, marriageability, and social belonging. As is the case across diverse cultural contexts, motherhood is commonly treated as a core marker of female identity, and women who delay or avoid childbearing are often perceived as selfish or emotionally incomplete (24–26). These internalized and socially reinforced expectations create intense psychological and social pressure to become, and remain, a mother. In contexts with strong familial ties, such as Libya, this pressure is intensified by cross-generational expectations and extended family obligations that compel women to prove their fertility soon after marriage.

One pronounced expression of fertility-related expectations revealed in our study is the pursuit of male offspring. In Libya, as in many parts of the Middle East and North Africa (MENA) and South Asia, repeated pregnancies are often driven by the desire for sons, who are viewed as essential to lineage continuity, family honor, and long-term social and financial security (27, 28). In patrilineal societies where inheritance and family name pass through the male line, sons are seen as markers of parental success and family resilience (29). This becomes particularly salient in contexts of conflict and weak social protections (30) where families turn to sons for economic stability and physical protection in the absence of institutional support (31). In such contexts, reproductive decisions are often shaped by existing structures that position sons as essential to family survival and status.

Religious interpretations, particularly conservative framings, further intensify these dynamics. While Islamic jurisprudence includes a spectrum of views, with many fatwas permitting contraception (32), our study indicated that conservative interpretations tend to dominate practice in Libya. A key finding of our study is that contraceptive access is often conditionally accepted when use can be framed through morally or religiously legitimate purposes. Providers often deny contraceptives to unmarried women or require proof of marriage, and many women, especially those perceived as too young or unmarried, face suspicion, moral judgment, or outright refusal when seeking contraceptive services. However, access may be more easily facilitated when contraceptive use aligns with religious or socially sanctioned purposes, for instance, to meet requirements for performing Umrah or in preparation for marriage. Similar patterns have been observed in other Muslim-majority and Christian-majority settings, where religious authority has shaped not only attitudes toward contraception, but also the conditions under which contraceptive use is considered legitimate or acceptable (33–35). In Libya, such conditioned provision contributes to a service environment in which reproductive rights are upheld inconsistently and access remains unreliable.

These social and religious pressures are not experienced in isolation. They are compounded by political and legal frameworks that cast reproductive services, particularly contraceptives, as suspicious or morally threatening. In Libya, where all sexual activity outside of marriage is criminalized under the Penal Code (36), access to contraception is often perceived as enabling illicit behavior, especially for unmarried women. It is also framed by some decision-makers as evidence of Western interference or as an attempt to undermine national identity. This politicization mirrors patterns documented in other postcolonial and conflict-affected settings, where reproductive governance constructs fertility as a national asset, a symbol of demographic strength, cultural continuity, and moral authority (37–39). In such contexts, efforts to limit or space births may be framed as undermining collective resilience, rather than fulfilling health needs. Health providers and civil society actors in Libya respond to this by using softer terminology such as “spacing” rather than “contraception,” aiming to reduce backlash while preserving services. However, this phrasing suggests the promise that childbearing is inevitable and merely delayed, not questioned or negotiated, which reinforces the expected biological role of women. It also potentially ties contraceptive use more closely to marriage and family formation, which could narrow who is recognized as an appropriate user of contraception. This may have important implications for adolescents and unmarried women, whose needs may fall outside socially sanctioned reproductive roles (40, 41).

The sociopolitical and religious pressures surrounding contraception in Libya unfold within a fragmented and under-resourced health system. Public facilities do not routinely stock contraceptives, which remain excluded from national procurement systems (10). As a result, many women rely on pharmacies or private clinics and often resort to secrecy and informal networks to obtain contraception. Our findings support previous evidence showing that, when formal systems fail to uphold reproductive rights, women adopt informal strategies to exercise agency, often at great personal cost (42). These acts of negotiation can be read both as resistance and compliance: secrecy enables women to avoid oppressive norms, but it also sustains the silence that allows those norms to persist (25). Secrecy also places providers in morally conflicted positions, in which they have to navigate between patient care and institutional constraints.

Despite these tensions, our findings suggest that attitudes toward family size are shifting. Participants cited financial insecurity, displacement, and political instability as reasons to limit or delay childbearing. This shift does not necessarily represent ideological rejection of traditional norms but rather a form of pragmatism, a recalibration of reproductive desires to fit changing realities. In other parts of the MENA region, economic pressure encourages a move toward smaller, gender-balanced families (28). In Libya, women spoke about stopping at two or three children or using contraception quietly after having one boy and one girl. These decisions reflect an emergent logic of adaptation rather than defiance.

The policy vacuum relegates family planning to a low priority in Libya, often overshadowed by security concerns and inconsistently addressed across humanitarian and development sectors. This silence has consequences, as it marginalizes women's reproductive needs within humanitarian and development agendas and reinforces a system in which women's health is governed by moral and political agendas (43).

Yet, we demonstrate that women are not passive recipients of policy; they act strategically to manage their reproductive lives, often under constraints and at risk. Supporting this agency requires more than a consistent supply of contraceptive commodities, it requires a reproductive justice approach that centers dignity, safety, and self-determination. Future programming must engage providers, families, and religious leaders in building inclusive, community-grounded models of care. Public procurement systems must incorporate modern contraceptives into essential medicines lists and provider training must address both technical competence and moral bias. Moreover, research must fill the gaps left by the absence of national surveillance: collecting reliable data on unmet need, user experience, and provider discretion is essential for designing accountable systems. In doing so, Libya can begin to transform the social and structural conditions under which reproductive choices are made, moving toward a framework where contraception is not only available, but meaningfully accessible and socially legitimized.

### 4.1 Limitations of the study

Although we engaged with participants from diverse urban settings, we did not include women from rural communities; they may experience even greater barriers to contraceptive access. Reliance on self-reported accounts may also have introduced recall or reporting bias, particularly given the sensitivity of discussing contraceptive use and reproductive intentions. The political and security context in Libya, including conflict that erupted in some of the study areas during data collection, created challenges in scheduling and rescheduling interviews and FGDs and may have affected the consistency and depth of some discussions. Moreover, ongoing government scrutiny of reproductive health and “morality” issues may have limited participants’ willingness to speak openly, further constraining some narratives. The lead researcher's position as a native Libyan Arabic speaker, extensive experience in SRH research in Libya, and direct involvement throughout data collection, translation, and analysis strengthened interpretive continuity across the dataset. However, as with all translated qualitative data, some linguistic nuance or contextual meaning may still have been lost in translation. While this study focused on Libya, its findings may be transferable to other fragile settings but should be interpreted with sensitivity to local variations. Future research could explore the perspectives of subgroups not represented here, such as adolescents, displaced women, and those living in remote areas, to capture a more comprehensive understanding of how contraceptive experiences and access dynamics may vary across forms of vulnerability not captured in the current study.

## 5 Conclusion

We demonstrate that contraceptive access cannot be understood through service availability alone, but must be examined through the wider social, religious, political, and structural conditions that shape contraceptive use in practice**.** In Libya, these overlapping pressures constrain women's reproductive choices in ways that make access conditional, morally scrutinized, and politically sensitive. Although some methods are formally available, access is often conditional and influenced by expectations of motherhood, provider discretion, legal ambiguity, and moral scrutiny. Contraceptives frequently are framed as morally suspect and politically disruptive and are often withheld or restricted on the basis of perceived social appropriateness rather than medical need. Women respond to these constraints through a range of strategies, many of which are discreet and informal. Providers also face limitations, working within systems that lack institutional support and expose them to social and political risk. The absence of clear national policy on contraception reflects a broader governance approach in which women's reproductive health is regulated through informal authority rather than protected as a right. These findings add to understandings of contraceptive access in fragile and politically restrictive settings and underscore the need for inclusive and context-sensitive policies that center women's lived experiences, reduce systemic barriers, and promote reproductive autonomy.

## Data Availability

The authors will provide de-identified data supporting the conclusions of this article upon reasonable written request.
